# Optimizing the Seahorse XF Mito Stress Test Workflow and Troubleshooting Notes: A Stepwise Protocol for HUVECs

**DOI:** 10.3390/metabo16020099

**Published:** 2026-01-28

**Authors:** Jingyi Wang, Yue Jiao, Jingzhe Li, Yanyan Ma, Changzhen Liu, Jing Yang

**Affiliations:** Experimental Research Center, China Academy of Chinese Medical Sciences, Beijing 100700, China; laoshuxiaozhi@126.com (J.W.); jiaoyue_medicine@163.com (Y.J.); lijingzhe@merc.ac.cn (J.L.); mayanyan@merc.ac.cn (Y.M.)

**Keywords:** Seahorse XF Pro, mitochondrial function, Mito Stress Test, HUVECs, protocol optimization, metabolic profiling, endothelial cells

## Abstract

This protocol details an optimized step-by-step procedure for performing the Seahorse XF Cell Mito Stress Test on human umbilical vein endothelial cells (HUVECs) using the Agilent Seahorse XF Pro Analyzer. Designed to address practical challenges often overlooked in standard manuals, the method preserves the native adherent state of HUVECs—a key *in vitro* model in vascular aging (VA) research—enabling real-time, label-free measurement of mitochondrial respiration and glycolytic function without cell detachment. The workflow is presented chronologically, covering instrument preparation, cell seeding, compound loading, assay execution, and post-assay normalization, with integrated notes and troubleshooting tips refined through hands-on experience based on the official manuals. This protocol aims to set up a detailed, rearranged standard workflow to improve experimental efficiency, reduce operator error, and support reproducible and well-organized metabolic profiling of HUVECs in aging and cardiovascular studies.

## 1. Introduction

As the global population ages, understanding mechanisms of age-associated pathologies like cardiovascular diseases (CVDs) is crucial [1,2]. Vascular aging (VA), a key driver of CVDs [3,4], involves the structural and functional decline of blood vessels, with endothelial cell (EC) dysfunction and mitochondrial impairment being central to its progression [5,6,7].

Human umbilical vein endothelial cells (HUVECs) serve as a well-established *in vitro* model for vascular biology due to their reproducible culture and relevance to age-related phenotypes [8,9]. Therefore, accurately assessing mitochondrial function in intact, adherent HUVEC monolayers—preserving their physiological context—is a critical experimental objective [10,11]. This requires a technique that is quantitative, non-invasive, and capable of real-time kinetic measurements.

**Figure 1:**
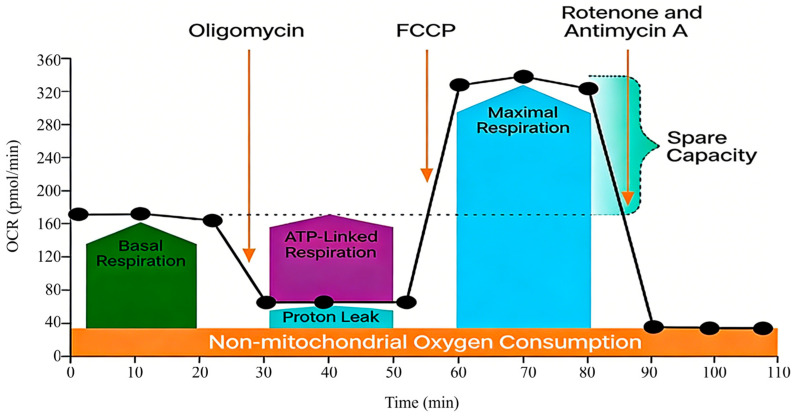
A schematic diagram of the Seahorse XF Cell Mito Stress Test Profile [18]. This schematic, adapted from the principle outlined in the Agilent Seahorse XF Cell Mito Stress Test Kit manual [18], visually summarizes the experimental workflow and the expected OCR response profile following sequential injections of oligomycin, Carbonyl Cyanide-4-(Trifluoromethoxy) Phenylhydrazone (FCCP), and rotenone/antimycin A. It serves as a foundational reference for understanding the assay logic within the context of this human umbilical vein endothelial cells (HUVEC) -optimized protocol. For additional details regarding the specific principles of this kit, please refer to the Agilent Product Brochure [18].

The Seahorse XF Extracellular Flux Analyzer addresses this need by enabling real-time, label-free measurement of the oxygen consumption rate (OCR) and extracellular acidification rate (ECAR) in adherent cells [12]. Its integrated Mito Stress Test allows for dynamic profiling of key mitochondrial parameters through sequential compound injections [13,14,15,16,17,18] (Figure 1).

While comprehensive user manuals [18] and published protocols [19,20] detail the general principles, their application to primary adherent cells like HUVECs often lacks cell-type-specific optimization for critical steps such as seeding density determination, Carbonyl Cyanide-4-(Trifluoromethoxy) Phenylhydrazone (FCCP) titration, and workflow scheduling. These practical nuances are paramount for assay robustness and reproducibility. The optimization steps detailed in this protocol were refined through experience gained from over five independent Seahorse XF Cell Mito Stress Test assays conducted in our laboratory.

## 2. Experimental Design

This protocol is structured into three sequential phases: (1) preparatory steps, including instrument warm-up, sensor cartridge hydration, and cell seeding; (2) assay execution, involving assay medium preparation, compound loading, and real-time OCR/ECAR measurement; and (3) post-assay processing, primarily protein quantification for data normalization.

**Figure 2:**
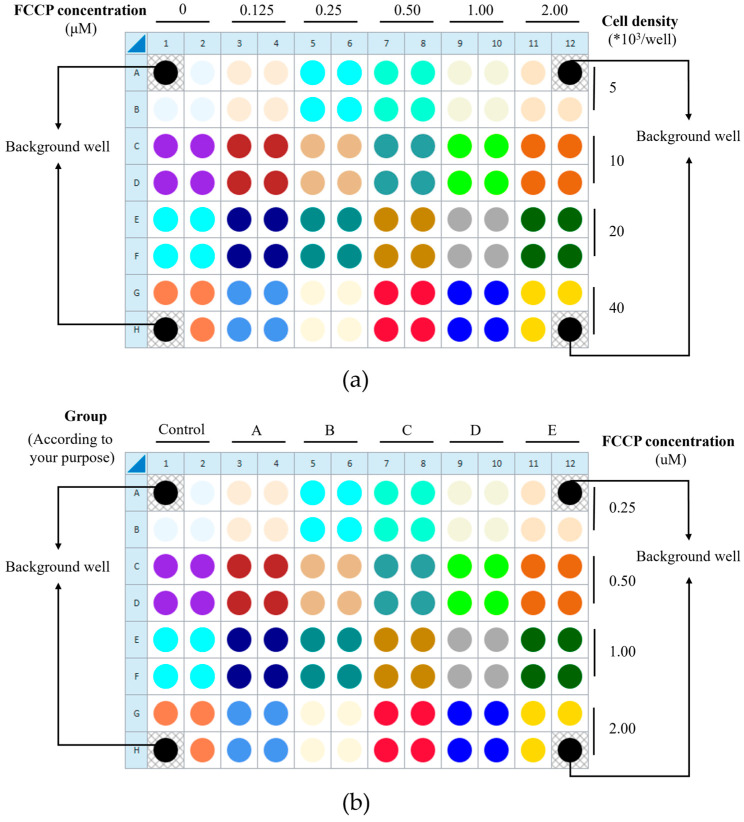
The orthogonal matrix design is to determine (**a**) the appropriate cell density and FCCP concentration, and (**b**) the factorial design. A1, A12, H1, and H12 are blank wells. (**a**) The orthogonal matrix design can be employed for initial optimization of the appropriate cell seeding density and FCCP concentration. In this design, each pair of adjacent columns (columns 1–2, 3–4, etc.) represents one FCCP concentration, allowing a maximum of six different concentrations to be tested per plate (the number of columns allocated can be adjusted as needed). Similarly, each pair of adjacent rows (A–B, C–D, etc.) corresponds to one cell density, enabling the evaluation of up to four densities per plate (the row arrangement can also be modified according to experimental requirements). In follow-up studies, different experimental groups may be arranged according to the research objectives (once the cell seeding density has been determined). Here, (**b**) illustrates the layout of different experimental groups (six groups in total) based on the previously optimized cell seeding density for this protocol as 1 × 10^4^ cells/well. The images presented are screenshots taken during the experimental operation of the operational interface, with subsequent annotation and illustrative processing added.

To ensure data quality, background correction wells (medium only) and within-plate technical replicates are incorporated. Successful execution with HUVECs requires preliminary optimization of two critical parameters: the cell seeding density and FCCP concentration. This protocol provides guidance using an orthogonal matrix design. For initial optimization, the orthogonal matrix design is recommended (Figure 2a). Once an appropriate cell density is determined, the plate layout can be adapted into a factorial design for efficient experimentation: each pair of adjacent columns can represent different experimental groups, while each pair of adjacent rows can represent different FCCP concentrations, enabling the simultaneous exploration of both variables (Figure 2b).

**Figure 3:**
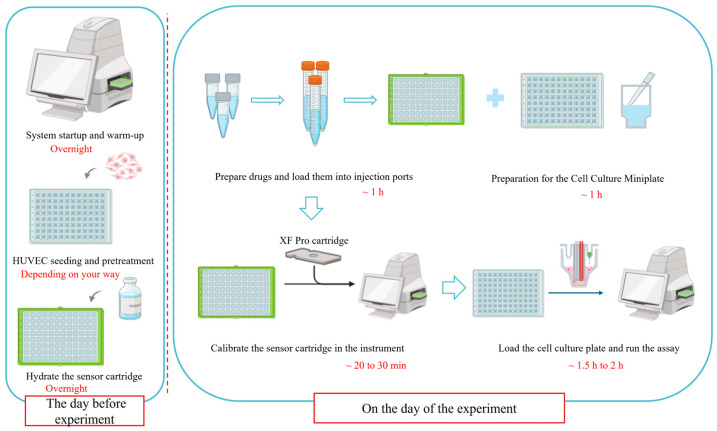
A schematic overview of the optimized Seahorse XF Cell Mito Stress Test workflow for HUVECs. Key stages and recommended time allocations are indicated. This figure was created by bioRender.com.

The entire workflow, with estimated timeframes for each major stage, is visually summarized in Figure 3. This schematic is intended to aid in planning and to highlight the chronological dependencies between steps.

To enhance robustness and reproducibility, this protocol emphasizes several optimized features: a defined strategy for parameter optimization, a detailed timeline and troubleshooting notes to prevent procedural errors, and a workflow checklist (Appendix A, Checklist of the Workflow) for critical step verification.

### 2.1. Materials

HUVECs (Shanghai Zhongqiaoxinzhou Biotech, Shanghai, China; Cat. no.: DFSC-EC-01).

Endothelial Cell Medium (ScienCell Research Kaboratories, Carlsbad, CA, USA; Cat. no.: 1001) including 500 mL Endothelial Cell Medium, 25 mL FBS, 5 mL ECGS, and 5 mL P/S solution.

Cell culture supplies including Trypsin (Gibco, Grand Island, NY, USA; Cat. no.: 25200-056), 1X PBS (Solarbio science & technology, Beijing, China; Cat. no.: P1020).

Angiotensin II (meilunbio, Dalian, China; Cat. no.: 4474-91-3).

Notoginsenoside R1 (Shanghai yuanye Bio-Technology Co., Ltd., Shanghai, China; Cat. no.: B21099).

Ginsenoside Rg1 (Shanghai yuanye Bio-Technology Co., Ltd., Shanghai, China; Cat. no.: B21057).

Resveratrol (Shanghai yuanye Bio-Technology Co., Ltd., Shanghai, China; Cat. no.: B20044).

Nuclease-free water (LABLEAD, Beijing, China; Cat. no.: D0055WJ).

96-well plate (Corning, Corning, NY, USA; Cat. no.: 3894).

Column Tissue & Cell Protein Extraction Kit (epizyme, Shanghai, China; Cat. no.: PC201plus) or 10X RIPA buffer (Millipore, Darmstadt, Germany; Cat. no.: 20-188).

BCA Protein Assay kit (Solarbio science & technology, Beijing, China; Cat. no.: PC0020).

Seahorse XF Cell Mito Stress Test Kit (Agilent Technologies, Santa Clara, CA, USA; Cat. no.: 103015-100) including oligomycin (powder), FCCP (powder), rotenone (powder), and antimycin A (powder).

Seahorse XF DMEM medium, pH 7.4, 500 mL (Agilent Technologies, Santa Clara, CA, USA; Cat. no.: 103575-100).

Seahorse XF 1.0 M glucose solution, 50 mL (Agilent Technologies, Santa Clara, CA, USA; Cat. no.: 103577-100).

Seahorse XF 100 mM pyruvate solution, 50 mL (Agilent Technologies, Santa Clara, CA, USA; Cat. no.: 103578-100).

Seahorse XF 200 mM glutamine solution, 50 mL (Agilent Technologies, Santa Clara, CA, USA; Cat. no.: 103579-100).

Seahorse XFe96/XF Pro FluxPak Mini (Agilent Technologies, Santa Clara, CA, USA; Cat. no.: 103793-100) including Agilent Seahorse XFe96 sensor cartridge, Agilent Seahorse XFe96 cell culture plate, and XF Calibrant Solution.

The specific brands and catalog numbers of consumables and reagents listed above represent the products validated in our laboratory for this protocol using HUVECs. While alternative products from other suppliers may be suitable, we emphasize that consistency within an experiment and the inclusion of appropriate internal controls are paramount for minimizing batch-to-batch or vendor-related variability and ensuring robust comparative analysis. Researchers adopting this protocol are encouraged to validate key reagents in their own experimental setting if alternatives are used.

### 2.2. Equipment

Agilent Seahorse Analyzer (Agilent Technologies, USA; Cat. no.: XF Pro).

Seahorse Wave Controller Software (version 10.1.0) (Agilent Technologies, Santa Clara, CA, USA).

Seahorse Wave Desktop Software (version 2.6.4) (Agilent Technologies, Santa Clara, CA, USA).

Clean bench (Airtech, Suzhou, China; Cat. no.: SW-CJ-1FD).

Non-CO_2_ incubator (Sunne, Shanghai, China; Cat. no.: SN-DH-36A).

Forma™ Steri-Cycle™ CO_2_ Incubator (Thermo Fisher Scientific, Waltham, MA, USA; Cat. no.: 371).

Plate reader for the BCA Protein Assay (BioTek, Winooski, VT, USA; Cat. no.: EON).

Centrifuge (Sigma, Munich, Germany; Cat. no.: 3K15).

## 3. Procedure

### 3.1. Cell Seeding: Two Experimental Strategies

Depending on the treatment paradigm, choose one of the following two strategies for preparing and seeding HUVECs into the Seahorse XFe96 cell culture microplate. Whichever strategy is chosen, ensure that the final cell confluence at the time of assay is within 50%~90% and that treatment durations are adjusted to avoid affecting cell viability and the metabolic baseline.

#### 3.1.1. Strategy A: Treatment Followed by Seeding

Culture and treat HUVECs with compounds or perform transfection in standard culture vessels (e.g., 6-well plates) according to your established protocols. On the day before the Seahorse assay, harvest the cells using trypsinization, centrifuge, resuspend in complete growth medium, and perform a cell count. Seed the cell suspension directly into the Seahorse cell culture microplate at the optimized density (see Section 3.2.3 and Figure 2a) and incubate overnight for attachment prior to the assay. In this protocol, we choose this strategy as standard.

#### 3.1.2. Strategy B: Seeding Followed by Treatment

Seed untreated HUVECs directly into the Seahorse microplate at the optimized density several days prior to the assay, allowing for full adhesion. Apply treatments (compounds, transfection, etc.) directly to the wells of the Seahorse cell culture plate. Pay particular attention to the duration of intervention to ensure it concludes at the desired point immediately before the assay. Proceed directly to the assay medium exchange and assay run.

### 3.2. Preparation of Cells and Cartridge Before the Formal Experiment (One Day Before Assay)

#### 3.2.1. Instrument Open and Startup

Turn on the Seahorse analyzer and allow it to connect, initialize, and warm up overnight (37 °C).

#### 3.2.2. Hydrate Cartridge and Pre-Warm XF Calibrant Solution

Hydrate the sensor cartridge by adding 200 µL of sterile water to all of the 96 sensor wells. Place the cartridge into a non-CO_2_ incubator set to 37 °C and allow it to hydrate undisturbed overnight with moats in the incubator. Then transfer 20 mL of the calibration solution into a 50 mL centrifuge tube. Incubate it overnight, also in a 37 °C non-CO_2_ incubator.

#### 3.2.3. Cells Preparation and Seeding

(1) Prepare Blank Controls: Pipette 200 μL of growth medium (complete ECM medium without cells) into the background correction wells of the Seahorse cell culture plate (i.e., Wells A1, A12, H1, and H12). (2) Prepare Cell Suspension: Harvest cells using a standard trypsinization procedure (HUVECs, usually using trypsin, are digested for around 1 min then finalized by adding complete ECM at 3 times the volume of trypsin). Resuspend the cell pellet in complete growth medium, perform a cell count, and dilute the suspension to the desired seeding density (for the first time we recommend from 5 × 10^3^ to 1 × 10^4^, 2 × 10^4^, and 4 × 10^4^ per well, according to Figure 2a). (3) Seed Cells and Microscopic Observation: Add 80 μL of the prepared cell suspension to the remaining experimental wells (except those mentioned above) in a biological safety cabinet at room temperature and leave undisturbed for 1 h. Visually inspect cell growth, confluence, and overall morphology using a phase-contrast microscope. (4) Incubate Cells: Transfer the cell plate into a standard cell culture incubator (37 °C, 5% CO_2_) and culture the cells overnight (usually 16 h) or for the desired duration. If culturing for more than 24 h, ensure the moats remain hydrated.

### 3.3. Preparation of Media and Cartridge Before the Formal Experiment (On the Day of Assay)

#### 3.3.1. Assay Medium Preparation

(1) Thaw/Equilibrate Components: Warm the L-glutamine solution in a water bath at 37 °C until the solution becomes fully clear and homogeneous. (2) Prepare Medium Formulation: Inside a clean bench or biosafety cabinet, prepare the complete assay medium by supplementing the required volume (97 mL) of Seahorse XF DMEM base medium with stock solutions of glucose (1 mL), sodium pyruvate (1 mL), and L-glutamine (1 mL) to achieve the following final concentrations independently: glucose 10 mM, sodium pyruvate 1 mM, and L-glutamine 2 mM. (3) Pre-warm Medium: Place the prepared assay medium in a non-CO_2_ incubator set to 37 °C or using a 37 °C water bath and allow it to equilibrate to temperature for at least 15~30 min prior to use.

#### 3.3.2. Sensor Cartridge Hydration

(1) Aspirate Sterile Water: Carefully aspirate sterile water from all wells. Pay extra attention to the handling techniques. (2) Replace Calibrant Solution: Replace it with the pre-warmed Seahorse XF Calibrant solution, ensuring the same volumes are used (200 µL per well). (3) Incubate and Equilibrate: Replace the probe plate carefully. Return the cartridge to the non-CO_2_, 37 °C incubator and allow it to equilibrate until the assay begins (usually around 60 min).

#### 3.3.3. Preparation of Compound Stocks and Working Solutions

(1) Kit Retrieval: Remove one foil pouch and the accompanying decapper tool from the Seahorse XF Cell Mito Stress Test Kit. (2) Reagent Reconstitution: Open the foil pouch and take out the 3 reagent tubes. Visually identify them by their cap colors: oligomycin (blue cap), FCCP (yellow cap), and rotenone/antimycin A (red cap). Prior to opening, centrifuge the tubes for 10 s at 1 × 10^4^ RPM to ensure all lyophilized powder is collected at the bottom. Use the provided decapper tool to carefully remove each cap. Reconstitute the contents of each tube by adding the specific volume of pre-warmed assay medium indicated before. Pipette up and down thoroughly (around 10 times) to ensure complete solubilization. These are now the compound stock solutions. (3) Working Solution Preparation: Prepare intermediate compound working solutions from the stocks for loading into the sensor cartridge. The following final in-well concentrations after 1:10 injections are commonly used as a starting point for most cell types: oligomycin (1.50 µM), FCCP (0.25 µM in our test) (usually the appropriate concentration will need to be found using the orthogonal matrix design method in Figure 2), rotenone/antimycin A (0.50 µM).

#### 3.3.4. Preparation for the Cell Culture Miniplate

(1) Retrieve and Inspect Cells: Remove the cell culture miniplate from the 37 °C, 5% CO_2_ incubator. Using a microscope, confirm that cells have reached the desired confluence (typically 50~90% for an ideal metabolic rate) and exhibit healthy morphology. (2) Change Assay Medium and Wash Cells: Working within a biosafety cabinet, carefully aspirate the complete growth medium from all wells of the miniplate. Make sure each well is left with about 20 µL of medium every time. Gently add 200 µL of the pre-warmed assay medium to each well and then remove 200 µL from all the wells. Repeat this wash procedure twice. (3) Add Assay Medium and Equilibrate: Gently add 160 µL of the pre-warmed assay medium to each well to ensure the final volume of each well equals 180 µL. Avoid disturbing the cell monolayer. Inspect the cell status via microscope and place the miniplate in a 37 °C, non-CO_2_ incubator for 45~60 min to allow the medium pH and temperature to stabilize prior to the assay.

#### 3.3.5. Cartridge Loading

(1) There are two strategies to decide from according to your experimental purpose: For the standard assay, load the working solutions of oligomycin, FCCP, and rotenone/antimycin A into injection ports A, B, and C, respectively. For a modified assay, if some other acute-effect compound needs to be tested, load the test compound into port A, then accordingly load the other three modulators (oligomycin, FCCP, and rotenone/antimycin A) into the next available ports in the original order (B, C, D). (2) Volume Calculation: The injection ports deliver a 1:10 dilution into the assay well. If each well contains 180 µL of medium, load the following volumes into the ports to account for the volume and sequential additions during the assay run: Port A 20 µL, Port B 22 µL, Port C 25 µL, Port D 27 µL. (For details about the addition methods, check the Notes and Troubleshooting part.)

#### 3.3.6. Instrument Setup and Assay Execution

(1) Initialize Assay Protocol: On the Seahorse XF Pro Analyzer software (Wave Pro 10.1.0), initiate a new assay. Select the default template for the “Agilent Seahorse XF Cell Mito Stress Test”. Proceed through the setup screens (e.g., click the right arrow on the “Groups” page) and finalize by clicking “Start Assay” on the Protocol page. Based upon your plate arrangement, you can set up your own group indication with different colors. (2) Calibrate the Sensor Cartridge: Place the utility plate containing the pre-hydrated and compound-loaded sensor cartridge onto the instrument tray. Click “Continue” to begin an automated calibration cycle, which typically requires around 20 min. Pay attention to the correct loaded direction. (3) Initiate the Stress Test: Upon completion of calibration, the instrument will prompt for the cell plate. Remove the utility plate, carefully replace it with the equilibrated Seahorse XFe96 Cell Culture Miniplate, and click “Continue” to commence the real-time OCR/ECAR measurements and sequential compound injections.

### 3.4. Post-Assay Protein Quantification for Data Normalization

Following the completion of the Seahorse Cell Mito Stress Test, the protein content from each well should be determined to normalize metabolic rates (OCR/ECAR) to cellular mass, thereby minimizing inter-well variability. The following protocol describes cell lysis and protein quantification using RIPA buffer (or, depending on your experimental potential, using a Column Tissue & Cell Protein Extraction Kit or other related kit) and a bicinchoninic acid (BCA) assay.

#### 3.4.1. Preparation of Lysis Reagent

Prior to the conclusion of the Seahorse assay, prepare fresh, ice-cold RIPA lysis buffer by diluting 10X RIPA stock solution with nuclease-free water and supplementing it with 100X HALT Protease and Phosphatase Inhibitor Cocktail according to the manufacturer’s instructions. Keep the prepared buffer on ice.

#### 3.4.2. Cell Lysis

Immediately after the assay, place the cell culture miniplate on ice or a pre-chilled plate cooler. Aspirate the assay medium completely from all wells. Gently wash each well twice with 200 µL of ice-cold phosphate-buffered saline (PBS), aspirating completely after each wash. Carefully add 50 µL of the prepared, ice-cold RIPA lysis buffer to each well of the 96-well plate. Wells containing only medium (i.e., background control Wells A1, A12, H1, and H12) should be processed identically to serve as assay-specific blanks. Incubate the plate on ice for 20 min with occasional gentle shaking or tilting to ensure complete coverage and efficient lysis.

#### 3.4.3. Protein Quantification via BCA Assay

Prepare the BCA working reagent by mixing Reagent A with Reagent B at a 50:1 ratio, as specified by the manufacturer. In a new and clean 96-well plate, set up a standard curve in duplicate using a serial dilution of bovine serum albumin (BSA) standards, covering an appropriate concentration range (e.g., 0~0.50 mg/mL). Transfer 20 µL of each protein lysate (from the former step) and each standard to the designated wells of the 96-well plate. (In the event that dilution is necessary, the relevant samples should be diluted either as directed in the kit instructions or in accordance with established laboratory practice.) Add 200 µL of the BCA working reagent to each well containing sample or standard. Cover the plate and incubate it at 37 °C for 15~30 min. After incubation, measure the absorbance of each well at 562 nm using a microplate reader.

#### 3.4.4. Data Analysis and Normalization

Generate a standard curve by plotting the average absorbance of the BSA standards against their known concentrations. Determine the protein concentration (in µg/µL) for each unknown sample using the linear regression equation derived from the standard curve. Calculate the total protein mass per original well by multiplying with the sample concentration (µg/µL) if dilution proceeded. These final protein yield values (in µg) will be used for the normalization of Seahorse metabolic rates in Wave software (version 2.6.4) or subsequent data analysis.

### 3.5. Seahorse Data Extraction and Normalization

Upon completion of the Seahorse assay, metabolic data (e.g., OCR and ECAR) can be exported from the instrument in multiple formats, including native Wave files, Excel spreadsheets, or files compatible with GraphPad Prism (version 9.0.0), depending on your needs.

#### 3.5.1. Data Processing with Wave Software

The Agilent Wave Desktop software product (version 2.6.4) provides integrated Report Generators for standard assay types (e.g., Mito Stress Test). These tools automatically calculate key bioenergetic parameters (such as basal respiration, ATP-linked respiration, and maximal respiration) from the raw data after they have been exported from the Wave file into a structured Excel worksheet.

#### 3.5.2. Normalization Procedure

To correct variations in cell number or biomass across wells, follow these steps to normalize the extracted data. Within the Wave software interface, navigate to the “Normalize” tab on the home page. Specify the normalization method by selecting either “Cell Number” or “Protein Amount.” Based upon our previous procedure, input the corresponding total protein quantity (in µg) calculated for each well from the post-assay BCA assay into the designated fields. Click Apply to execute the normalization. The software will adjust all metabolic rate data (e.g., OCR, ECAR) accordingly. The Normalize tab can be toggled on or off to instantly compare the normalized and non-normalized data views for quality control and an assessment of normalization’s impact.

## 4. Results

The following results are presented to demonstrate the workflow execution and data output style attainable by strictly following this optimized protocol. 

### 4.1. OCR, ECAR, and Energy Map

**Figure 4:**
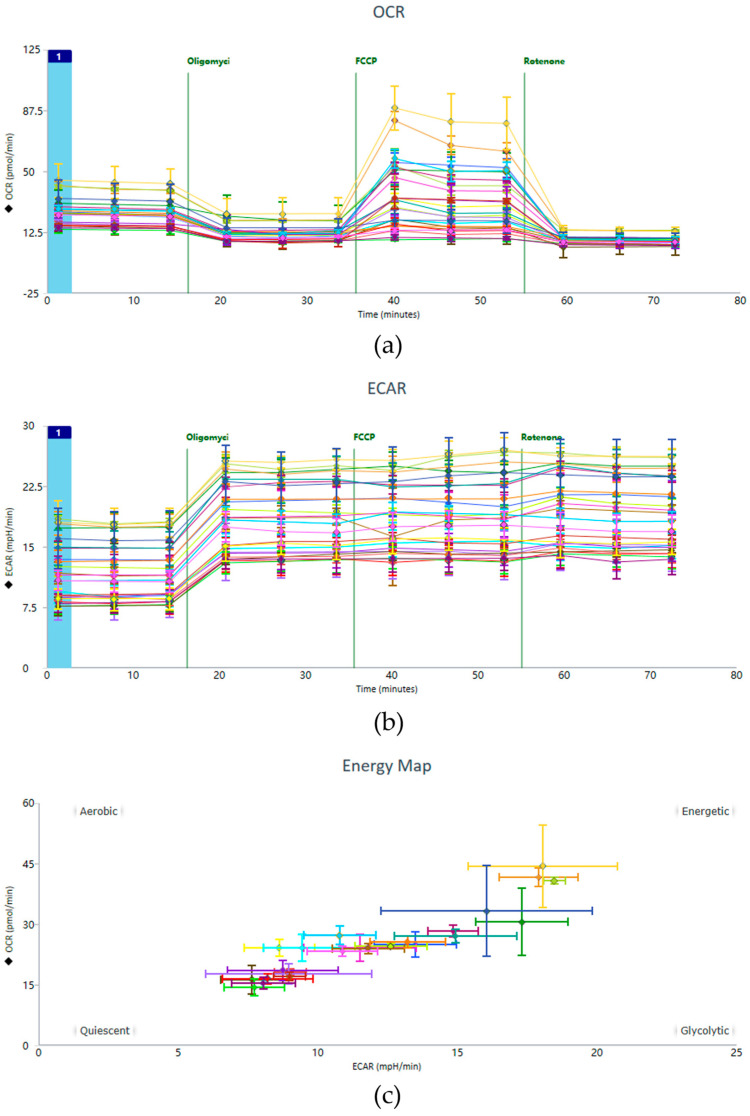
Exemplary raw data including the (**a**) OCR, (**b**) ECAR, and (**c**) Energy Map. This figure presents representative experimental results, depicting the data panel generated following the implementation of the optimized operational protocol.

Representative data from the Seahorse XF Cell Mito Stress Test, including OCR and ECAR profiles, are shown in Figure 4. These profiles illustrate the characteristic responses of HUVEC monolayers to sequential injections of metabolic modulators (oligomycin, FCCP, and rotenone/antimycin A).

### 4.2. Plate Map, Group List, and Data View

**Figure 5:**
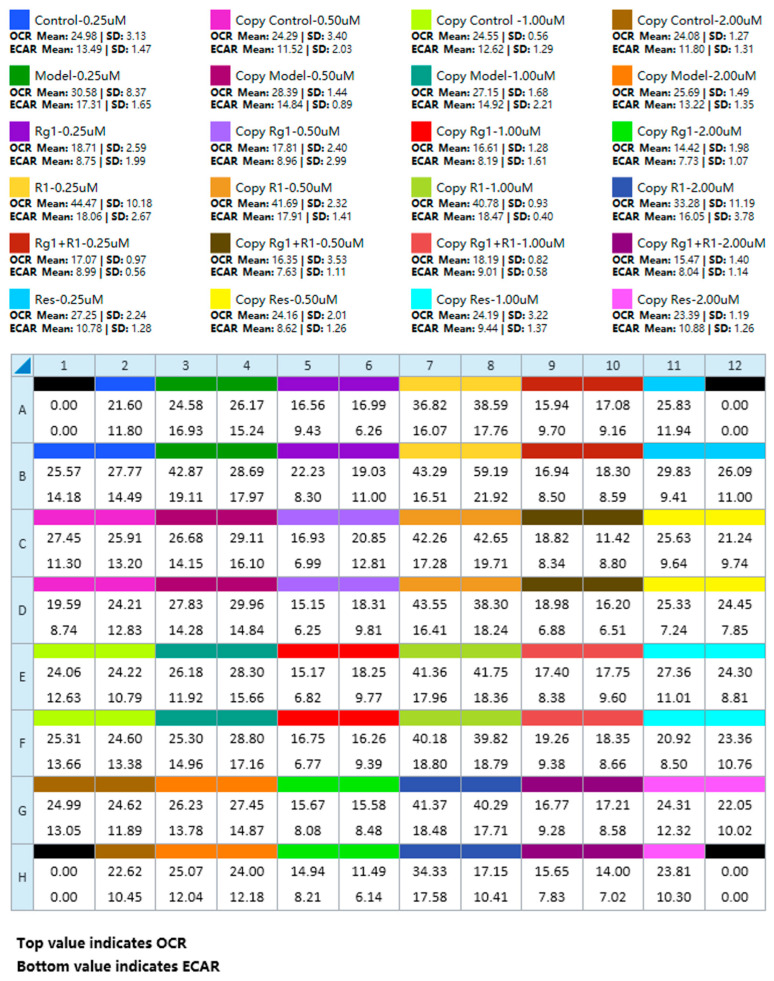
A summary plate map and the corresponding data view layout for a multi-group experiment. This figure presents representative experimental results, depicting the data panel generated following the implementation of the optimized operational protocol.

Figure 5 provides a summary plate map and the corresponding data view layout for a multi-group experiment, serving as a practical template for experimental setup and primary data visualization.

It is important to re-emphasize that the data shown here are exemplary. Their purpose is to validate the protocol’s robustness rather than to support specific biological conclusions. While detailed statistical analysis (e.g., using software such as GraphPad Prism) is essential for hypothesis-driven research, it falls outside the core scope of this methodological article, which focuses on optimizing the experimental workflow to ensure reproducible and efficient raw data acquisition.

## 5. Notes for Easily Missed Details and Troubleshooting

- Do not overlook the step of instrument opening and startup, as insufficient warming up may lead to errors in test results. Additionally, forgetting this step could cause other subsequent processing steps to be completed while the instrument remains inadequately warmed up, resulting in prolonged waiting time for cells and potentially compromising their condition, thereby affecting the results.
- It is important to use proper and gentle techniques whenever handling the probe plate to prevent probe damage.
- For initial experiments, it is still recommended to use the orthogonal matrix design to determine an optimal concentration in the Seahorse cell culture plate to define a suitable cell density and FCCP concentration.
- The seeding area per well of the XFe96 cell culture plate is 0.106 cm^2^, which is 40% of the seeding area per well of a standard 96-well cell culture plate (this can be appropriately adjusted and optimized based on your previous experience with seeding in 96-well plates). Before seeding cells, the optimal seeding density should be determined as it varies for different cell types; it is typically optimized within the range of 5 × 10^3^~4 × 10^4^ cells/well. If this is not suitable, please adjust the cell density range. According to previous articles, aging HUVEC cell densities could be taken as examples for a related range (Table 1) using other types of Seahorse Analyzer.

**Table 1:** Recommendations for HUVEC number/well based upon experimental purposes in accordance with related references to Mito Stress Tests.

No.	Seahorse Model	Cell Number/Well	Culture Duration	Study Purpose
1	XF24	1 × 10^4^	48 h Starvation 24 h Prevention 24 h	Atovastatin on mitochondrial energy metabolism [13]
2	XF24	2 × 10^4^	28 h Adherence 4 h Drug coincubation 24 h	Advanced glycation end products on mitochondrial energy [21]
3	XF24	3 × 10^4^	30–32 h Adherence 6–8 h Starvation 24 h	ALDH2 on mitochondrial oxygen reserve capacity in ECs [22]
4	XF24	6.25 × 10^4^	48 h (Details not mentioned)	VEGF on endothelial metabolism [23]

**Figure 6:**
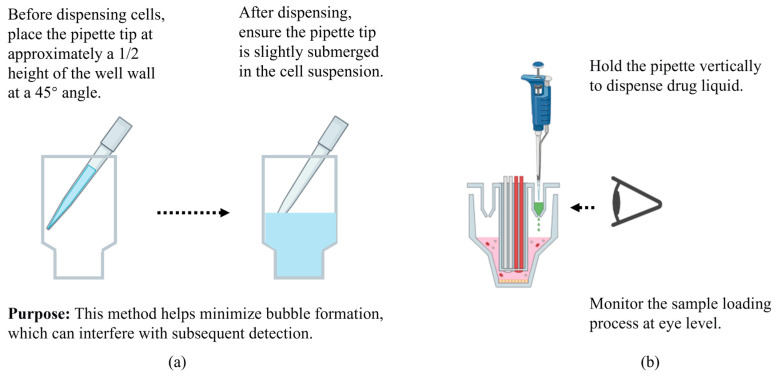
Addition techniques for cell seeding (**a**) and drug loading (**b**). This figure was created by bioRender.com.

- It is important to note that if the seeding volume is calculated as 80 µL/well, the cell suspension density should be calculated as 1 × 10^4^ cells (as an example)/80 µL/well = 1.25 × 10^5^ cells/mL. This is an easily forgotten step.
- L-Glutamine solution must be frozen at −20 °C. As all assay reagents should be prepared freshly before use, it is recommended to aliquot the reagent into EP tubes immediately upon receipt, freezing 1 mL per tube. When needed, thaw an aliquot and ensure it is thoroughly mixed before use.
- The Seahorse DMEM (corresponding to the catalog number mentioned in the Materials Section 2.1.) has been pre-adjusted for pH. After the XF supplement is added, no further pH adjustment is required. If other products are used, an additional step to adjust the pH is necessary.
- When preparing drug solutions, it is recommended to use extended-length pipette tips for mixing. Otherwise, due to the narrow tube diameter, thorough mixing may not be achieved, or spillage may easily occur during the mixing process.
- One foil pouch includes enough compounds for just one test instance. Leftover compounds after the test cannot be frozen and are not suitable for the next test instance.
- When handling cells, place the tip at a 45° angle against the well wall at mid-height before dispensing, and keep it slightly submerged in the suspension afterward. This combined approach effectively minimizes bubble formation to prevent interference with downstream detection (Figure 6a).

- Before cells are loaded onto the instrument for detection, the degassing and equilibration time should preferably not exceed 60 min, as longer durations may affect the detection results.
- When washing the cells, pay attention to the handling of the four blank (background) wells. Pipette tips should be changed after adding compounds to each column to prevent cross contamination.
- Notes on Drug Addition Technique: The accompanying drug-loading auxiliary plate can be used according to personal preference (different orientations allow for dosing into different well positions). Since improper use of the auxiliary plate may result in incomplete delivery of the drug solution, an alternative method is to insert a multichannel pipette vertically into the drug ports and inject the solution slowly in one continuous motion without pausing, minimizing bubble formation. When observing the delivery of the solution (Figure 6b), avoid lifting the plate arbitrarily to prevent spillage; instead, view it at eye level to check the wells. Whenever possible, use a multichannel pipette with accurate volume calibration to reduce dispensing errors.
- During the cell washing process, you may use a calibrated well to perform a sampling test to detect and roughly estimate the remaining liquid volume in the wells. It is not strictly necessary to achieve exactly 180 µL but try to keep it as close as possible.
- You may first complete drug treatment, then digest and centrifuge the cells before seeding them onto the Seahorse cell culture plate. Alternatively, you can seed the cells directly into the Seahorse cell culture plate before performing the treatment until the assay begins. However, since the two approaches result in different total culture durations on the Seahorse plate, the cell seeding density should be adjusted accordingly.
- Based on our experimental study, using Ang II as an *in vitro* cell aging inducer for 60 h and using Notoginsenoside R1, Ginsenoside Rg1, and Resveratrol as prevention treatments for 48 h, the final cell density adopted was 1 × 10^4^ cells per well, with an FCCP concentration of 0.25 μM. However, specific parameters such as the cell density and concentration of FCCP or other compounds should still be adjusted according to actual experimental conditions or further optimized based on the relevant literature within the overall framework provided.
- To prevent common errors and ensure a smooth, reliable workflow, a comprehensive, phase-specific checklist is provided in Appendix A, Checklist of the Workflow. This checklist serves as a core tool for systematic quality control and workflow organization. We strongly recommend printing it out and using it to perform a step-by-step verification at each key stage of the protocol. Actively consulting and completing this checklist is crucial for preventing oversight and ensuring strict procedural adherence. Furthermore, researchers are encouraged to adapt and expand this template based on their specific experimental conditions, thereby enhancing its utility as a personalized instrument to safeguard consistency and reproducibility.

## Data Availability

The original contributions presented in the study are included in the article. Further inquiries can be directed to the corresponding authors.

## Abbreviations

CVDs	Cardiovascular diseases
VA	Vascular aging
EC	Endothelial cell
HUVECs	Human Umbilical Vascular Endothelial Cells
OCR	Oxygen consumption rate
ECAR	Extracellular acidification rate
FCCP	Carbonyl Cyanide-4-(Trifluoromethoxy) Phenylhydrazone
BCA	Bicinchoninic acid
PBS	Phosphate-buffered saline
BSA	Bovine serum albumin

## Appendix A

### Checklist of the Workflow

**Checklist for Seahorse Mito Cell Test Assay Workflow**					
Date:		Operator:		Instrument Status:	
Memo:					
Process Stage	Specific Steps	Key Points for Details			Check Board
Part I The day before the assay	1. Instrument Open and Startup	Make sure the analyzer connects, initializes, and warms up overnight (37 °C).			
	2. Hydrate Cartridge	Add 200 μL sterile water per well, incubate at 37 °C in a non-CO_2_ incubator overnight, and handle with care to avoid probe damage.			
	3. Pre-warm XF Calibrant Solution	Transfer 20 mL to a 50 mL centrifuge tube and incubate overnight at 37 °C in a non-CO_2_ incubator. (PS: Step 3 and step 4 can be completed simultaneously.)			
	4. Cell Preparation and Seeding	Prepare blank controls with medium in designated wells; harvest, count, and dilute cells to recommended density (e.g., 5 × 10^3^–4 × 10^4^/well); seed 80 µL suspension into experimental wells; let settle at room temperature for 1 h, then microscopically inspect before transferring to a 37 °C, 5% CO_2_ incubator for overnight culture (~16 h), keeping moats hydrated if extended.			
Part II On the Day of Assay	1. Assay Medium Preparation	Supplement XF DMEM base medium (97 mL) with glucose (1 mL), sodium pyruvate (1 mL), and L-glutamine (1 mL) to final concentrations of 10 mM, 1 mM, and 2 mM, respectively, in a biosafety cabinet; pre-warm at 37 °C in a non-CO_2_ incubator for 15~30 min before use.			
	2. Sensor Cartridge Hydration	Aspirate hydration water and replace with 200 µL pre-warmed XF Calibrant per well; incubate in non-CO_2_ incubator at 37 °C for ~60 min before assay.			
	3. Preparation of Compound Stocks and Working Solutions	Using a single-use Mito Stress Test Kit, reconstitute the three compounds (oligomycin, FCCP, rotenone/antimycin A) with pre-warmed assay medium. Prepare working solutions targeting final in-well concentrations (e.g., 1.50 µM, 0.25 µM *, 0.50 µM after 1:10 injection), noting that the optimal FCCP concentration requires prior titration.			
	4. Preparation of the Cell Culture Miniplate	Confirm cell confluence and health via microscope, **gently** wash cells twice with pre-warmed assay medium, then add 160 µL medium per well (final 180 µL) and equilibrate in a 37 °C non-CO_2_ incubator for 45~60 min.			
	5. Cartridge Loading (Select One Way)	Standard assay: Oligomycin (Port A 20 µL), FCCP (Port B 22 µL), and rotenone/antimycin A (Port C 25 µL).			
		Modified assay: Other test compound (Port A 20 µL), oligomycin (Port B 22 µL), FCCP (Port C 25 µL), and rotenone/antimycin A (Port D 27 µL).			
	6. Instrument Setup and Assay Execution	Initialize the “Mito Stress Test” protocol in the software, calibrate the loaded sensor cartridge (around 20 min), then replace with the cell plate to start real-time measurements and compound injections. PS: Note the loading direction.			

* This table streamlines and summarizes the key optimized steps, with critical procedures highlighted in red to facilitate user verification during procedure execution. Users may also consult the official instrument-specific protocols based on their laboratory’s Seahorse model at https://www.agilent.com.cn/zh-cn/product/cell-analysis/how-to-run-an-assay, accessed on 20 January 2026.

**Recommendation:** We sincerely recommend that the process start at around 4 P.M. the day before the assay to allow for enough preparation and a well-organized time schedule. According to our time record, Part I takes about 1~1.5 h, and Part II takes around 3.5~4.5 h till measurement starts for all the samples. The whole measurement process takes about 1.5 h. Based on the actual workflow, the procedure has been optimized by grouping identical steps together to minimize operational errors and by reorganizing the sequence according to execution speed. This adjustment aims to prevent prolonged cell incubation in the non-CO_2_ incubator prior to the assay, thereby reducing the risk of result inaccuracies.
